# Lung ultrasound in quantifying lung water in septic shock patients

**DOI:** 10.1186/cc14220

**Published:** 2015-03-16

**Authors:** L De Geer, A Oscarsson, M Gustafsson

**Affiliations:** 1 Linkoping University Hospital, Linkoping, Sweden

## Introduction

Quantification of lung ultrasound (LUS) artifacts (B-lines) is used to assess pulmonary congestion in emergency medicine and cardiology [1,2]. We investigated B-lines in relation to extravascular lung-water index (EVLWI) from invasive transpulmonary thermodilution in septic shock patients. Our aim was to evaluate the role of LUS in an intensive care setting.

## Methods

Twenty-one patients admitted with septic shock to a general ICU underwent LUS of eight zones, four per hemithorax, within 24 hours after ICU admission. EVLWI was calculated simultaneously by transpulmonary thermodilution using a pulse-contour continuous cardiac output system, and NT-proBNP and clinical data were collected. Two physicians blinded to other data independently quantified the number of B-lines. Spearman's rho was used to test the correlation of B-lines to EVLWI and clinical data, and linear regression and Bland- Altman analysis were used to assess the agreement between B-lines and EVLWI. Interobserver variability was tested using Bland-Altman analysis and intraclass correlation coefficient (ICC).

## Results

Fourteen patients (67%) were male, the median age was 62 years (IQR 55 to 68) and eight (38%) patients had cardiac comorbidities. In median, SAPS 3 was 64 (IQR 60 to 74), ICU length of stay was 3 days (IQR 2 to 8) and seven patients (33%) died within 30 days of ICU admission. All patients were mechanically ventilated and treated according to guidelines [3]. The median number of B-lines was 15 (IQR 10 to 30) and the median (IQR) NT-proBNP, EVLWI and oxygenation index (OI) were 7,800 ng/l (3,690 to 15,050), 11 ml/kg (IQR 8 to 18) and 9.2 (5.7 to 15.7), respectively. None of the characteristics differed significantly between survivors and nonsurvivors. The number of B-lines correlated to EVLWI (ρ = 0.45, *P *= 0.04; *r*^2 ^= 0.20, *P *= 0.04), but not to NT-proBNP (ρ = -0.42, *P *= 0.06), OI (ρ = 0.25, *P *= 0.31) or ICU length of stay (ρ = 0.14, *P *= 0.57). On Bland-Altman analysis, mean differences and 95% limits of agreements between B-lines and EVLWI was 4.9 (-14.5 to 24.5), and 5.9 (-3.5 to 15.3) when assessing observer agreement. The ICC between methods was 0.52 (95% CI = -0.17 to 0.81) and 0.90 (95% CI = 0.73 to 0.92) between observers.

## Conclusion

LUS non-invasively and user-independently quantifies lung water in concordance with, but does not replace, invasive measurements. Further studies are needed establish the role of LUS as a monitoring and diagnostic tool in septic shock patients.
